# Relapsing Fever Spirochete in Seabird Tick*,* Japan

**DOI:** 10.3201/eid1509.090459

**Published:** 2009-09

**Authors:** Ai Takano, Maki Muto, Akiko Sakata, Yumiko Ogasawara, Shuji Ando, Nozomu Hanaoka, Miyako Tsurumi, Fumio Sato, Noboru Nakamura, Hiromi Fujita, Haruo Watanabe, Hiroki Kawabata

**Affiliations:** National Institute of Infectious Diseases, Tokyo, Japan (A. Takano, M. Muto, A. Sakata, Y. Ogasawara, S. Ando, N. Hanaoka, H. Watanabe, H. Kawabata); Gifu University, Gifu, Japan (A. Takano, H. Watanabe, H. Kawabata); Yamashina Institute for Ornithology, Chiba, Japan (M. Tsurumi, F. Sato, N. Nakamura); Ohara General Hospital, Fukushima, Japan (H. Fujita)

**Keywords:** Borrelia, relapsing fever, Carios, seabird, bacteria, spirochetes, letter

**To the Editor:** Tick-borne relapsing fever (TBRF) is caused by infection with spirochetes belonging to the genus *Borrelia*. We previously reported a human case of febrile illness suspected to be TBRF on the basis of serologic examination results; the vector most likely was a genus *Carios* tick that had fed on a seabird colony (*1*). However, surveillance of ticks in the area did not identify *Borrelia* spp. in any of the *Carios* ticks sampled (*2*). In 2007 and 2008, a borreliosis investigation was conducted on Kutsujima Island (35.71′N, 135.44′E) because a bird-associated tick, genus *Carios*, inhabits this island. During the investigation, 77 *Carios* ticks (55 nymphs, 11 adult males, and 11 adult females) were collected from colonies of seabirds: Swinhoe's storm petrel (*Oceanodroma monorhis*) and streaked shearwater (*Calonectris leucomelas*)*.* Identification of tick species as *C. sawaii* was based on tick morphology and *rrs* gene sequence analysis of the tick mitochondrion DNA (*2*). Total DNA was extracted from the ticks by using a DNeasy Tissue Kit (QIAGEN, Germantown, MD, USA). For the detection of *Borrelia* DNA, PCR designed was based on the flagellin gene (*flaB*) according to Sato et al. (*3*). To check for contamination and amplicon carryover, we used blank tubes as a negative control for each experiment. Of 77 *C. sawaii* ticks that were positive by PCR of tick genes (*2*), 25 (14 nymphs, 6 adult males, 5 adult females) were positive for *Borrelia* DNA by PCR of *flaB*.

To characterize the *Borrelia* spp., we sequenced amplified fragments of the *flaB* gene and the 16S ribosomal RNA (*16SrRNA*) gene of *Borrelia* spp. in a tick and compared the results with those of representative *Borrelia* spp. The primers BflaPBU and BflaPCR (*3*) for *flaB* and the 4 PCR primers (Technical Appendix) for *16SrRNA* were used for direct sequencing and/or amplification. DNA sequence (GenBank accession no. AB491928) of a 294-bp amplified fragment of *flaB* showed the following nucleotide similarities with those of *Borrelia* spp.: *B. turicatae* (98.98%), *B. parkeri* (98.30%), *Borrelia* sp. Carios spiro-1 (98.64%), and *Borrelia* sp. Carios spiro-2 (98.30%). DNA sequence (GenBank accession no. AB491930) of a 1,490-bp amplified fragment of *16SrRNA* showed the following nucleotide similarities with those of *Borrelia* spp.: *B. turicatae* (99.60%), *B. parkeri* (99.53%), and *Borrelia* sp. Carios spiro-2 (99.45%). *Borrelia* sp. Carios spiro-1 and Carios spiro-2, which were recently identified in *C. kelleyi* in the United States, have been classified into TBRF *Borrelia* (*4*,*5*). The *Borrelia* sp. found in this study, designated as *Borrelia* sp. K64, was closely related to *B. turicatae* but was distinct from other TBRF *Borrelia* spp. (Technical Appendix).

To observe *Borrelia* spp. in tick tissues, we performed an indirect fluorescence assay (IFA) according to methods described by Fisher et al. (*6*), with minor modifications. A tick that was negative by PCRs of *flab* and *16SrRNA* was used as a negative control. The IFA of the tick salivary gland and midgut was conducted by using acetone for fixation, goat anti-*Borrelia* sp. polyclonal immunoglobulin (Ig) G (1:100; KPL, Inc., Gaithersburg, MD, USA) as the primary antibody, and Alexa fluor 488-labeled rabbit antigoat IgG (1:200, Invitrogen, Carlsbad, CA, USA) as the secondary antibody. Our analysis showed a spirochete, which was stained by anti-*Borrelia* spp. antibody, in salivary gland and midgut tissue (Technical Appendix). However, no spirochetes were detected by IFA in the negative control (data not shown).

We also attempted to isolate *Borrelia* spp. from tick specimens by using Barbour-Stoenner-Kelly medium (*7*). The motility of *Borrelia*-like organisms in the medium was initially observed by using dark-field microscopy. The *Borrelia-*like organisms were identified as *Borrelia* sp. K64 by sequencing of PCR-amplified fragments of *flaB* and *16SrRNA* genes from the cultured medium. However, these *Borrelia* organisms were found for only 2 weeks after inoculation (data not shown).

The vertebrate reservoir hosts of TBRF *Borrelia* are usually rodents but can be a variety of other animals (*8*). Although competence as a reservoir has not been determined for birds, infection of an owl with a TBRF *Borrelia* sp. has been reported (*9*). The vertebrate host of the spirochete has not yet been determined. Given our results, it is possible that seabirds are potential vertebrate hosts for *Borrelia* spp.

In Japan, relapsing fever is a neglected infectious disease because it was not reported during 1956–1998 (*10*). In this study, we detected a *Borrelia* sp. in *C. sawaii*, and the spirochete we characterized is closely related to *B. turicatae*. Although the human health implications of infections caused by *Borrelia* spp. are not yet known, the findings from this study should contribute to the epidemiologic investigation of TBRF in Japan.

## Supplementary Material

Technical Appendix Relapsing Fever Spirochete in Seabird Tick, Japan
