# Supplementary material for: Relapsing Fever Spirochete in Seabird Tick, Japan
Source: Emerg Infect Dis. 2009 Sep;15(9):1528–30. doi: 10.3201/eid1509.090459 (PMC2819885; doi:10.3201/eid1509.090459)
Supplement: Technical Appendix — Relapsing Fever Spirochete in Seabird Tick, Japan [file 09-0459_Techapp-s1.pdf]

Relapsing Fever Spirochete in Seabird Tick, Japan

Technical Appendix

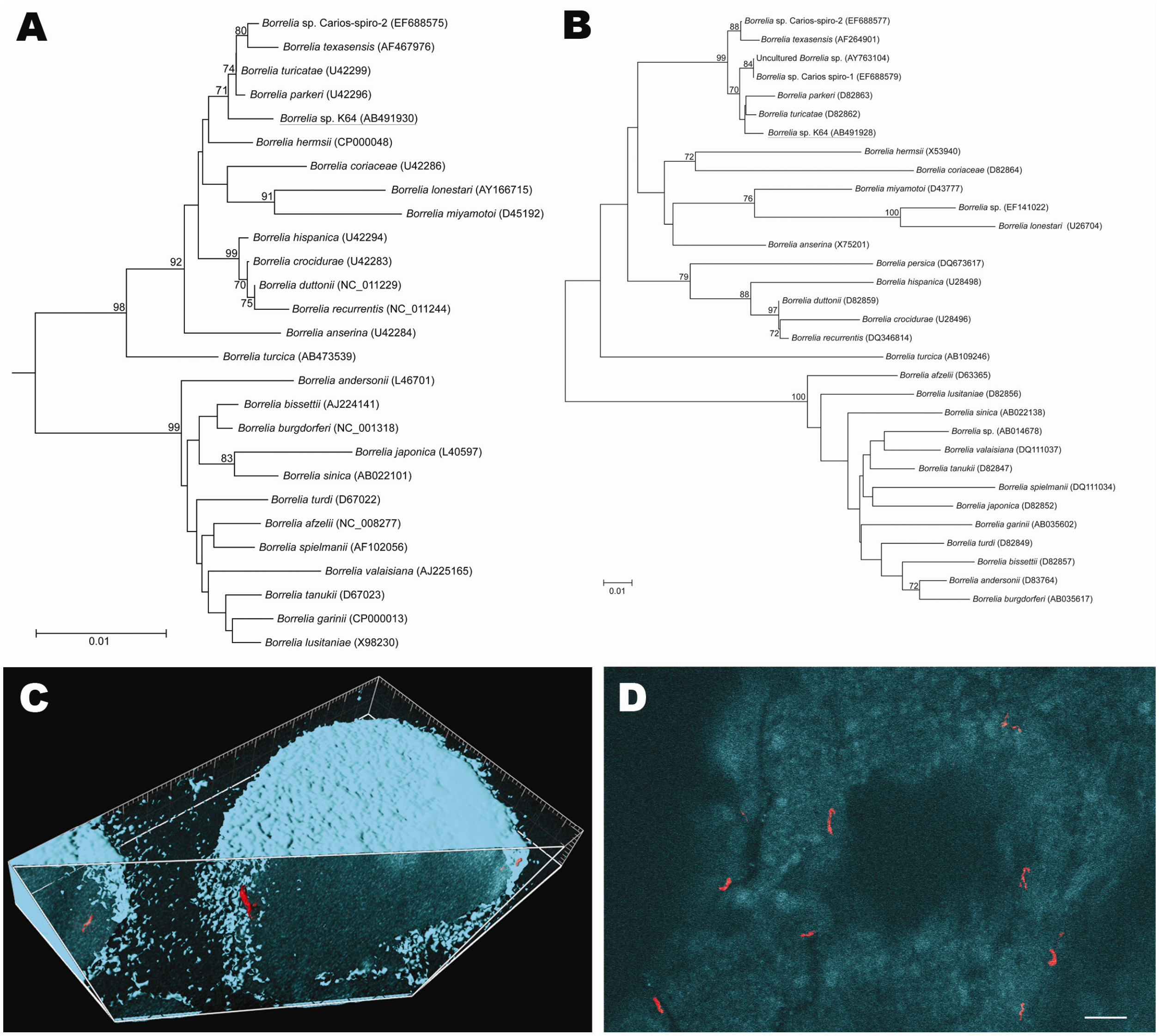

Phylogenetic analysis based on A) the 16S ribosomal RNA gene (*16SrRNA*) and B) flagellin gene of *Borrelia* spp. Representative *Borrelia* spp. were used. GenBank accession numbers are in parentheses. For the amplification and direct sequencing of *16SrRNA*, the 4 PCR primers rrs-F1 (5′-ATAACGAAGAGTTTGATCCTGGCT-3′), rrs-F2 (5′-GGTGTAAGGGTGGAATCTGTTG-3′), rrs-R3 (5′-TTTCGTGACTCAGCGTCAGT-3′), and rrs-R4 (5′-AAAGGAGGTGATCCAGCCRCACT-3′) were used. These primers were designated by DNA sequence alignment of 5 of *16SrRNA* (GenBank accession nos.

NC_001318 [*B. burgdorferi*], CP000048 [*B. hermsii*], CP000013 [*B. garinii*], NC_008277 [*B. afzelii*], and NC_011229 [*B. duttonii*]). The phylogenetic trees were constructed by using neighbor-joining methods, and a bootstrap test was carried out according to the Kimura 2-parameter distances method. The percentage of replicate trees in which the associated taxa were clustered together in the bootstrap test (1,000 replicates) was calculated. More than 80% of the phylogenetic branches were supported by bootstrap analysis. The scale bar indicates the percentage of sequence divergence. All positions containing alignment gaps and missing data were eliminated in the pairwise sequence comparisons (pairwise deletion). Phylogenetic analyses were conducted by using MEGA4 (www.megasoftware.net). *Borrelia* sp. K64 (underlined) was detected from *Carios sawaii* tick samples in this study. *Treponema pallidum* (GenBank accession no. NC_000919), *Spirochaeta americana* (AF373921) and *Cristispira* sp. (U42638) were used as outgroups (not shown) for phylogenetic analysis based on *16SrRNA*. Panels C and D show spirochetes (red) in the acinus of a salivary gland (3D surface and cutaway projection by confocal microscopy, smallest scale increment = 1μm) (C) and midgut (immunofluorescence assay, scale bar = 10 μm) (D) from an unfed *C. sawaii* tick. The DNA fragment of *Borrelia* sp. K64 was detected in another side of the salivary gland and in a part of midgut of the same tick, respectively.
